# The Study Variety of Conformal Kinematics

**DOI:** 10.1007/s00006-022-01227-x

**Published:** 2022-07-18

**Authors:** Bahar Kalkan, Zijia Li, Hans-Peter Schröcker, Johannes Siegele

**Affiliations:** 1 Department of Mathematics, Van Yuzuncu Yil University, Van, Turkey; 2 KLMM, Academy of Mathematics and Systems Science, Chinese Academy of Sciences, Beijing, China; 3 Department of Basic Sciences in Engineering Sciences, University of Innsbruck, Innsbruck, Austria

**Keywords:** Simple motion, Study variety, Study quadric, Null quadric, Four quaternion representation, Factorization, Primary 15A66, Secondary 15A67, 20G20, 51B10, 51F15

## Abstract

We introduce the Study variety of conformal kinematics and investigate some of its properties. The Study variety is a projective variety of dimension ten and degree twelve in real projective space of dimension 15, and it generalizes the well-known Study quadric model of rigid body kinematics. Despite its high dimension, co-dimension, and degree it is amenable to concrete calculations via conformal geometric algebra (CGA) associated to three-dimensional Euclidean space. Calculations are facilitated by a four quaternion representation which extends the dual quaternion description of rigid body kinematics. In particular, we study straight lines on the Study variety. It turns out that they are related to a class of one-parametric conformal motions introduced by Dorst in (Math Comput Sci 10:97–113, 2016, 10.1007/s11786-016-0250-8). Similar to rigid body kinematics, straight lines (that is, Dorst’s motions) are important for the decomposition of rational conformal motions into lower degree motions via the factorization of certain polynomials with coefficients in CGA.

## Introduction

Conformal Geometric Algebra (${\text {CGA}}$) is a commonly used algebra for describing conformal displacements [1, 4]. This paper is dedicated to the study of conformal kinematics of three-space from an algebraic and geometric viewpoint. Our aim is to describe a construction of a point model of conformal kinematics, similar to the well-known Study quadric model of rigid body kinematics, c.f. [15, Chapter 11], to investigate some of its properties, and to relate it to existing knowledge on ${\text {CGA}}$. The Study quadric model is based on the representation of the group ${\text {SE}}(3)$ of rigid body displacements by dual quaternions $\mathbb {D}\mathbb {H}$. Rigid body displacements correspond to points of a quadric $\mathcal {Q}$ in $\mathbb {P}(\mathbb {D}\mathbb {H}) = \mathbb {P}^7(\mathbb {R})$, the *Study quadric.* Its equation is given by the condition that the dual quaternion norm is real. We will define the *Study variety* $\mathcal {S}$ of conformal kinematics as subvariety in the projective space $\mathbb {P}^{15}(\mathbb {R})$ over the even sub-algebra ${\text {CGA}} _+$ of Conformal Geometric Algebra of three-dimensional Euclidean geometry. Its ideal encodes the well-known spinor conditions, which demand that the products of an element $r \in {\text {CGA}} _+$ with its reverse from the left or from the right be real. Our treatment is based on a *four quaternion representation* for elements of the 16-dimensional algebra ${\text {CGA}} _+$ which allows transparent computations and highlights the similarities to the “two quaternion representation” of ${\text {SE}}(3)$ via dual quaternions.

The group ${\text {SE}}(3)$ of rigid body displacements is of high importance in robotics and mechanism science and, because the dual quaternions $\mathbb {D}\mathbb {H}$ form a sub-algebra of ${\text {CGA}} _+$, is naturally embedded into conformal kinematics. While Clifford algebra approaches to rigid body kinematics are, of course, well-known, c.f. [14] or [1, Chapter 7], we observe that some typical approaches to problems of robotics or mechanism science via $\mathbb {D}\mathbb {H}$ have not yet been generalized to conformal kinematics. Examples include the geometry of the Study quadric and its relation to space kinematics in the sense of [11, 12, 15], the study of constraint varieties [2, 13, 16], and the factorization theory of motion polynomials [6, 10]. We feel that it is worth extending or generalizing these concepts to ${\text {CGA}} _+$. This not only teaches us about rigid body kinematics but also adds value to ${\text {CGA}} _+$, it provides structure and new insight.

In particular, an important motivation when writing this text is to prepare the ground for a factorization theory of “spinor polynomials”, a conformal generalization of motion polynomials. On the kinematics level, the factorization corresponds to a decomposition of conformal motions with rational trajectories into the product of motions of lower degrees. The basic building blocks are linear polynomials. They correspond to straight lines on the Study variety $\mathcal {S}$ and, as we shall see in Sect. 4, they parametrize a class of motions introduced by Dorst in [3] as the exponential of 2-blades (the wedge product of two vectors).

After recalling some well-known facts about the conformal geometric algebra associated to 3D space in Sect. 2, the subsequent Sect. 3 features an investigation of the Study variety $\mathcal {S}$ based on a four quaternion representation of ${\text {CGA}} _+$. There we also introduce the null quadric $\mathcal {N}$, a quadric in $\mathbb {P}^{15}$ which we consider important for the geometric explanation of certain kinematic phenomena. One instance of this can be found in Sect. 4 where we characterize straight lines through the identity on $\mathcal {S}$, proof that they are precisely the elementary motions investigated in [3], and relate the intersection points with $\mathcal {N}$ to Dorst’s generation from two vectors. Combining the concepts and methods developed in previous sections with standard factorization algorithms for Clifford algebras [9] already allows computing multiple decompositions of conformal motions given by generic spinor polynomials into products of simple motions. We provide one example in Sect. 5.

## Conformal Geometric Algebra and Dual Quaternions

We follow the conventions of [1, Chapter 8] or [17] for constructing the conformal model of *n*-dimensional Euclidean space but we specialize to the case $n = 3$ immediately. We pick an orthonormal basis $\{e_1,e_2,e_3,e_+,e_-\}$ of $\mathbb {R}^{4,1}$ with the properties

$$\begin{aligned} e_1^2 = e_2^2 = e_3^2 = e_+^2 = 1,\quad e_-^2 = -1. \end{aligned}$$

The geometric product of two basis vectors is defined to be anticommutative:

$$\begin{aligned} e_ie_j = -e_je_i \quad \text {for pairwise different}\quad i,j \in \{1,2,3,+,-\}. \end{aligned}$$

By linear extension, it generates the real algebra ${\text {CGA}}$. It is customary, to replace $e_+$, $e_-$ by $e_0$, $e_\infty$ via

$$\begin{aligned} e_o = \tfrac{1}{2}(e_- - e_+),\quad e_\infty = e_- + e_+ \end{aligned}$$

and to write $e_{ij}$ for $e_ie_j$, $e_{ijo\infty }$ for $e_ie_je_oe_\infty$ etc. The *reverse* $\widetilde{e}_\ell$ of $e_\ell$ is obtained by inverting the order of elements in $\ell$, that is, $\widetilde{e}_{\ell _1 \cdots \ell _n} = e_{\ell _n \cdots \ell _1}$. The *grade* of the basis element $e_\ell$ with $\ell \subset \{1,2,3,o,\infty \}$ is the cardinality of $\ell$.

A general element of ${\text {CGA}}$ can be written as

1 $$\begin{aligned} a = \sum _{\ell \subset \{1,2,3,o,\infty \}} a_\ell e_\ell , \quad \text {where}\quad a_\ell \in \mathbb {R}. \end{aligned}$$

Its reverse is $\widetilde{a} = \sum a_\ell \widetilde{e}_\ell$. The even subalgebra ${\text {CGA}} _+$ of ${\text {CGA}}$ consists of all real linear combinations of even grade basis elements. If all basis elements in (1) are of grade one, *a* is called a *vector*. The scalar product of two vectors is precisely the scalar product in ${\mathbb {R}}^{4,1}$. Later, we will also need the wedge product of two vectors, which is defined as $a \wedge b \,{:}{=}\,ab - a \cdot b$. Note that $a \wedge a = 0$. A Euclidean point $(x_1,x_2,x_3)$ is represented in ${\text {CGA}}$ as the vector

2 $$\begin{aligned} x=e_0+x_1e_1+x_2e_2+x_3e_3 +\frac{x_1^2+x_2^2+x_3^2}{2} e_\infty , \end{aligned}$$

the point at infinity is represented by $e_\infty$. Vectors $n_1e_1+n_2e_2+n_3e_3+de_\infty$ where the coefficient of $e_o$ is zero represent planes with normal $n=(n_1,n_2,n_3)$ and oriented distance *d*/||*n*|| to the origin. All other vectors $a=\alpha (e_0+m_1e_1+m_2e_2+m_3e_3 + 1/2(m_1^2+m_2^2+m_3^2-\sigma ) e_\infty )$ in ${\text {CGA}}$ represent spheres with midpoint $(m_1,m_2,m_3)\in \mathbb {R}^3$ and radius $r=\sqrt{\sigma }=\sqrt{a\widetilde{a}/\alpha ^2}$. If $\sigma$ is negative, the sphere has an imaginary radius. It is natural to view spheres with zero radius as Euclidean points with spheres of radius zero, that is, real multiples of vectors in (2).

A rotor is often defined as an element of ${\text {CGA}} _+$ satisfying $a\widetilde{a} = \widetilde{a}{a} = 1$. Rotors form a double-cover of the group of direct conformal displacements of conformally closed three-dimensional Euclidean space $\mathbb {R}^3 \cup \{\infty \}$ (c.f. [1, Chapter 8.4]). We relax the rotor condition to $a\widetilde{a} = \widetilde{a}a = \pm 1$, thus arriving at the algebra’s spin group, a double-cover of the group of conformal displacements which is isomorphic to ${\text{ SO }}(4,1)$. In order to get rid of the representation ambiguities, we consider the spin group modulo the real multiplicative group $\mathbb {R}^\times$. We call its elements *homogeneous spinors.*

A homogeneous spinor is represented by an element $a \in {\text {CGA}} _+$ which satisfies

3 $$\begin{aligned} a\widetilde{a} = \widetilde{a}a \in \mathbb {R}{\setminus } \{0\} \end{aligned}$$

This is our preferred interpretation as it gives rise to a model of conformal kinematics as projective variety. As the representation of homogeneous spinors is only unique up to non-zero scalar multiples, we can interpret homogeneous spinors as points in ${\text {CGA}} _+$ modulo the real multiplicative group $\mathbb {R}^\times$. This turns the *vector space* ${\text {CGA}} _+$ into the *projective space* $\mathbb {P}({\text {CGA}} _+)$. As the group of even-graded elements ${\text {CGA}} _+$ is of dimension 16 as a real vector space, we have $\mathbb {P}({\text {CGA}} _+) = \mathbb {P}^{15}(\mathbb {R})$. The conformal group ${\text{ SO }}(4,1)$ is embedded as a subvariety given by the equations arising from $a\widetilde{a} = \widetilde{a}a \in \mathbb {R}$ minus the variety given by $a\widetilde{a} = \widetilde{a}a = 0$. If $a \in {\text {CGA}} _+$ satisfies (3), we call $a\widetilde{a} = \widetilde{a}a$ the *norm of* *a*. As usual, equivalence classes of non-zero scalar multiples are denoted by square brackets, i.e., $[a] = [-a] = [2a]$ or $[1] = \mathbb {R}{\setminus } \{0\}$ etc. (Beware, this notation does not distinguish between the equivalence class [1] and a reference to a bibliography item! Context will tell what is meant.)

Above construction is similar to the description of rigid body kinematics via dual quaternions $\mathbb {D}\mathbb {H}$ (c.f. [8] or [15, Section 9.3]). The real algebra of dual quaternions is generated from the basis elements

$$\begin{aligned} \mathbf {i},\ \mathbf {j},\ \mathbf {k},\ \varepsilon ,\ \mathbf {i}\varepsilon = \varepsilon \mathbf {i},\ \mathbf {j}\varepsilon = \varepsilon \mathbf {j},\ \mathbf {k}\varepsilon = \varepsilon \mathbf {k}\end{aligned}$$

by the relations

$$\begin{aligned} \mathbf {i}^2 = \mathbf {j}^2 = \mathbf {k}^2 = \mathbf {i}\mathbf {j}\mathbf {k}= -1\quad \text {and}\quad \varepsilon ^2 = 0. \end{aligned}$$

It is easy to verify that the dual quaternions are contained in ${\text {CGA}} _+$ by the identifications

4 $$\begin{aligned} \mathbf {i}\mapsto -e_{23},\quad \mathbf {j}\mapsto e_{13},\quad \mathbf {k}\mapsto -e_{12},\quad \text {and}\quad \varepsilon \mapsto e_{123\infty }. \end{aligned}$$

The norm of the dual quaternion $a = a_0 + a_1\mathbf {i}+ a_2\mathbf {j}+ a_3\mathbf {k}+ \varepsilon (a_4 + a_5\mathbf {i}+ a_6\mathbf {j}+ a_7\mathbf {k})$ is

$$\begin{aligned} a\widetilde{a} = \widetilde{a}a = a_0^2 + a_1^2 + a_2^2 + a_3^2 + 2\varepsilon (a_0a_4 + a_1a_5 + a_2a_6 + a_3a_7) \end{aligned}$$

and the real norm condition reduces to

5 $$\begin{aligned} a_0a_4 + a_1a_5 + a_2a_6 + a_3a_7 = 0. \end{aligned}$$

The group of dual quaternions of non-zero norm modulo $\mathbb {R}^\times$ is isomorphic to ${\text {SE}}(3)$. Its elements can be mapped bijectively onto the points of the *Study quadric* $\mathcal {Q}\subset \mathbb {P}^7(\mathbb {R})$ given by Equation (5) minus the points of the *null cone* $\mathcal {C}\subset \mathbb {P}^7(\mathbb {R})$ given by the vanishing condition of the norm’s real part

$$\begin{aligned} a_0^2 + a_1^2 + a_2^2 + a_3^2 = 0. \end{aligned}$$

As explained in Sect. 1, this construction is relevant for the geometry and algebra of ${\text {SE}}(3)$ and has applications in mechanism science and robotics. In the next section, we extend it to the conformal group.

## Study Variety and Null Quadric

The counterpart of the Study quadric $\mathcal {Q}\subset \mathbb {P}^7$ in conformal kinematics is the *Study variety* $\mathcal {S}$, a ten-dimensional projective variety in $\mathbb {P}^{15}$ of degree twelve. It is complemented with the *null quadric* $\mathcal {N}$ which, in contrast to the null cone of the dual quaternion model of ${\text {SE}}(3)$, is a regular quadric. This is quite important, as $SV {\setminus } \mathcal {N}$ consists of two disjoint components that can be thought of as direct and indirect (orientation preserving and orientation reversing) conformal displacements. For the sake of a transparent derivation of equations we suggest a *four quaternion representation* of ${\text {CGA}} _+$. It naturally extends the dual (“two”) quaternion representation of ${\text {SE}}(3)$.

### Four Quaternion Representation

We distribute the 16 real coordinates of ${\text {CGA}} _+$ into four groups of four coordinates. The first group contains the coefficients of 1, $-e_{23}$, $e_{13}$, and $-e_{12}$, that is, it is a quaternion $r_0$ in the sense of (4). The second group contains the coefficients of basis elements whose index set contains $\infty$ but not *o*. With $\varepsilon _1 \,{:}{=}\,e_{123\infty }$, they are

$$\begin{aligned} \varepsilon _1 = e_{123\infty },\ \mathbf {i}\varepsilon _1 = -e_{23} \varepsilon _1 = e_{1\infty },\ \mathbf {j}\varepsilon _1 = e_{13} \varepsilon _1 = e_{2\infty },\ \mathbf {k}\varepsilon _1 = -e_{12} \varepsilon _1 = e_{3\infty }. \end{aligned}$$

We write the second group as $r_1\varepsilon _1$ with a quaternion $r_1$. Note that $\varepsilon _1$ here equals the dual unit $\varepsilon$ in (4) so that $r_1$ is the dual part of a dual quaternion. The third group contains the coefficients of basis elements whose index set contains *o* but not $\infty$. With $\varepsilon _2 \,{:}{=}\,e_{123o}$ they are

$$\begin{aligned} \varepsilon _2 = e_{123o},\quad \mathbf {i}\varepsilon _2 = -e_{23} \varepsilon _2 = e_{1o},\quad \mathbf {j}\varepsilon _2 = e_{13} \varepsilon _2 = e_{2o},\quad \mathbf {k}\varepsilon _2 = -e_{12} \varepsilon _2 = e_{3o}. \end{aligned}$$

We write this as $r_2\varepsilon _2$ with a quaternion $r_2$. The fourth group contains the remaining coefficients. Their respective index sets contain both, $\infty$ and *o*:

$$\begin{aligned} \varepsilon _1\varepsilon _2 = e_{\infty o},\quad \mathbf {i}\varepsilon _1\varepsilon _2 = -e_{23} \varepsilon _1\varepsilon _2 = -e_{23\infty o},\\ \mathbf {j}\varepsilon _1\varepsilon _2 = e_{13} \varepsilon _1\varepsilon _2 = e_{13\infty o},\quad \mathbf {k}\varepsilon _1\varepsilon _2 = -e_{12} \varepsilon _1\varepsilon _2 = -e_{12\infty o}. \end{aligned}$$

We write this as $r_3\varepsilon _1\varepsilon _2$ with a quaternion $r_3$.

Now, an element $q \in {\text {CGA}} _+$ has a unique representation as $q = r_0 + r_1\varepsilon _1 + r_2\varepsilon _2 + r_3\varepsilon _1\varepsilon _2$ with quaternions $r_0$, $r_1$, $r_2$, $r_3$. Note that $\widetilde{\varepsilon _1} = \varepsilon _1$, $\widetilde{\varepsilon _2} = \varepsilon _2$ but $\widetilde{\varepsilon _1\varepsilon _2} = -2 - \varepsilon _1\varepsilon _2$. In order to further simplify reversion, we make the change of basis $\varepsilon _1\varepsilon _2 \mapsto \varepsilon _3$ where

$$\begin{aligned} \varepsilon _3 \,{:}{=}\,\varepsilon _1\varepsilon _2 + 1 \end{aligned}$$

so that $\widetilde{\varepsilon _3} = -\varepsilon _3$. Note that $\varepsilon _3 = e_\infty \wedge e_o$, while $\varepsilon _1\varepsilon _2 = e_{\infty o}$. Again, there exist unique quaternions $q_0 = r_0 - r_3$, $q_1 = r_1$, $q_2 = r_2$, $q_3 = r_3$ such that $q = q_0 + q_1\varepsilon _1 + q_2\varepsilon _2 + q_3\varepsilon _3$. This we call the *four quaternion representation* of *q*.

Reversion in the four quaternion representation reads as

6 $$\begin{aligned} \widetilde{q} = \widetilde{q_0} + \widetilde{q_1}\varepsilon _1 + \widetilde{q_2}\varepsilon _2 - \widetilde{q_3}\varepsilon _3. \end{aligned}$$

The product of $p = p_0 + p_1\varepsilon _1 + p_2\varepsilon _2 + p_3\varepsilon _3$ and $s = s_0 + s_1\varepsilon _1 + s_2\varepsilon _2 + s_3\varepsilon _3$ is easily inferred from the multiplication Table 1 for $\varepsilon _1$, $\varepsilon _2$, and $\varepsilon _3$ and the fact that quaternions commute with $\varepsilon _1$, $\varepsilon _2$, and $\varepsilon _3$. We have

7 $$\begin{aligned} \begin{aligned} ps&= (p_0s_0 - p_1s_2 - p_2s_1 + p_3s_3) \\&\quad + (p_1(s_0 + s_3) + (p_0 - p_3)s_1)\varepsilon _1 \\&\quad + (p_2(s_0 - s_3) + (p_0 + p_3)s_2)\varepsilon _2 \\&\quad + (p_0s_3 + p_1s_2 - p_2s_1 + p_3s_0)\varepsilon _3. \end{aligned} \end{aligned}$$

**Table 1:** Multiplication table for $\varepsilon _1$, $\varepsilon _2$, and $\varepsilon _3$

	$\varepsilon _1$	$\varepsilon _2$	$\varepsilon _3$
$\varepsilon _1$	0	$\varepsilon _3 - 1$	$\varepsilon _1$
$\varepsilon _2$	$-\varepsilon _3 - 1$	0	$-\varepsilon _2$
$\varepsilon _3$	$-\varepsilon _1$	$\varepsilon _2$	1

### Ideal of the Study Variety

The Study quadric of rigid body displacements is the projective variety in $\mathbb {P}^7 = \mathbb {P}(\mathbb {D}\mathbb {H})$ defined by the condition (5) that its points have real norm. When generalizing this to ${\text {CGA}} _+$ we have to take into account that $q\widetilde{q} \ne \widetilde{q}q$ in general so that the proper definition reads:

#### Definition 1

The Study variety $\mathcal {S}$ of conformal kinematics is the projective variety in $\mathbb {P}^{15} = \mathbb {P}({\text {CGA}} _+)$ defined by the condition that both $q\widetilde{q}$ and $\widetilde{q}q$ are real.

A projective point $[q] \in \mathcal {S}$ describes a conformal displacement unless $q\widetilde{q} = \widetilde{q}q = 0$. We will have a closer look at the algebraic and geometric implications of this in Sect. 3.3. The generating equations of the Study variety are obtained by plugging $p = q$, $s = \widetilde{q}$ and $p = \widetilde{q}$, $s = q$ into (7):

8 $$\begin{aligned} \begin{aligned} q\widetilde{q}&= (q_0\widetilde{q}_0 - q_1\widetilde{q}_2 - q_2\widetilde{q}_1 - q_3\widetilde{q}_3) \\&\quad + (q_1(\widetilde{q}_0 - \widetilde{q}_3) + (q_0 - q_3)\widetilde{q}_1)\varepsilon _1 \\&\quad + (q_2(\widetilde{q}_0 + \widetilde{q}_3) + (q_0 + q_3)\widetilde{q}_2)\varepsilon _2 \\&\quad - (q_0\widetilde{q}_3 - q_1\widetilde{q}_2 + q_2\widetilde{q}_1 - q_3\widetilde{q}_0)\varepsilon _3, \end{aligned} \quad \begin{aligned} \widetilde{q}q&= (\widetilde{q}_0q_0 - \widetilde{q}_1q_2 - \widetilde{q}_2q_1 - \widetilde{q}_3q_3) \\&\quad + (\widetilde{q}_1(q_0 + q_3) + (\widetilde{q}_0 + \widetilde{q}_3)q_1)\varepsilon _1 \\&\quad + (\widetilde{q}_2(q_0 - q_3) + (\widetilde{q}_0 - \widetilde{q}_3)q_2)\varepsilon _2 \\&\quad + (\widetilde{q}_0q_3 + \widetilde{q}_1q_2 - \widetilde{q}_2q_1 - \widetilde{q}_3q_0)\varepsilon _3. \end{aligned}\nonumber \\ \end{aligned}$$

In order to write this more succinctly, we define

9 $$\begin{aligned} S(f,g) \,{:}{=}\,f\widetilde{g} + g\widetilde{f}, \end{aligned}$$

and ${{\,\mathrm{v}\,}}(f) \,{:}{=}\,\frac{1}{2}(f-\widetilde{f})$, the *vector part*, for quaternions *f*, *g*. With $f = f_0 + f_1\mathbf {i}+ f_2\mathbf {j}+ f_3\mathbf {k}$ and $g = g_0 + g_1\mathbf {i}+ g_2\mathbf {j}+ g_3\mathbf {k}$ we have $S(f,g) = 2(f_0g_0 + f_1g_1 + f_2g_2 + f_3g_3)$ so that $S(f,g) = 0$ is actually the Study condition for the dual quaternion $f + \varepsilon g$, compare with (5). In particular, $S(f,g)$ is bilinear in the real coefficients of *f* and *g*. Moreover, we have $S(f,g) = S(g,f) = S(\widetilde{f},\widetilde{g}) = \widetilde{f}g + \widetilde{g}f$. Using this notation, we can re-write the equations in (8) as

10 $$\begin{aligned} \begin{aligned} q\widetilde{q}&= (q_0\widetilde{q}_0 - S(q_1,q_2) - q_3\widetilde{q}_3) \\&\quad + (S(q_0,q_1) - S(q_1,q_3))\varepsilon _1 \\&\quad + (S(q_0,q_2) + S(q_2,q_3))\varepsilon _2 \\&\quad - 2({{\,\mathrm{v}\,}}(q_0\widetilde{q_3}) - {{\,\mathrm{v}\,}}(q_1\widetilde{q_2}))\varepsilon _3, \end{aligned} \quad \begin{aligned} \widetilde{q}q&= (\widetilde{q}_0q_0 - S(q_1,q_2) - \widetilde{q}_3q_3) \\&\quad + (S(q_0,q_1) + S(q_1,q_3))\varepsilon _1 \\&\quad + (S(q_0,q_2) - S(q_2,q_3))\varepsilon _2 \\&\quad + 2({{\,\mathrm{v}\,}}(\widetilde{q_0}q_3) + {{\,\mathrm{v}\,}}(\widetilde{q_1}q_2))\varepsilon _3. \end{aligned}\nonumber \\ \end{aligned}$$

Note that the coefficients of $\varepsilon _1$ and $\varepsilon _2$ are real while the coefficients of $\varepsilon _3$ are vectorial quaternions. The constant coefficients are the same and real as well. Thus, the ideal ${\mathcal {I}}$ of the Study variety is generated by the vanishing conditions of the coefficients of $\varepsilon _1$, $\varepsilon _2$, and $\varepsilon _3$. We will denote an ideal generated by polynomials $f_1$, $f_2,\ldots , f_n$ by $\langle f_1,f_2,\ldots ,f_n \rangle$. By a slight abuse of this notation, we can define

11 $$\begin{aligned} {\mathcal {I}}\,{:}{=}\,&\langle S(q_0,q_1), S(q_1,q_3), S(q_0,q_2), S(q_2,q_3), \nonumber \\ {}&{{\,\mathrm {v}\,}}(q_0\widetilde{q_3}) - {{\,\mathrm {v}\,}}(q_1\widetilde{q_2}), {{\,\mathrm {v}\,}}(\widetilde{q_0}q_3) + {{\,\mathrm {v}\,}}(\widetilde{q_1}q_2) \rangle . \end{aligned}$$

Note that the last two “generators” are vectorial and actually represent six real polynomials. We can get rid of them by using identities like

$$\begin{aligned} 2{{\,\mathrm{v}\,}}(q_0\widetilde{q_3}) = S(q_0, \mathbf {i}q_3)\mathbf {i}+ S(q_0, \mathbf {j}q_3)\mathbf {j}+ S(q_0, \mathbf {k}q_3)\mathbf {k}, \end{aligned}$$

and thus arriving at

12 $$\begin{aligned} {\mathcal {I}} \,{:}{=}\,&\langle S(q_0,q_1), S(q_1,q_3), S(q_0,q_2), S(q_2,q_3),\nonumber \\&S(q_0,\mathbf {i}q_3)-S(q_1,\mathbf {i}q_2), S(q_0,\mathbf {j}q_3)-S(q_1,\mathbf {j}q_2), S(q_0,\mathbf {k}q_3)-S(q_1,\mathbf {k}q_2),\nonumber \\&S(q_0,q_3\mathbf {i})+S(q_1,q_2\mathbf {i}), S(q_0,q_3\mathbf {j})+S(q_1,q_2\mathbf {j}), S(q_0,q_3\mathbf {k})+S(q_1,q_2\mathbf {k}) \rangle \end{aligned}$$

We see that the ideal ${\mathcal {I}}$ of the Study variety is generated by ten bilinear polynomials. Using computer algebra software it can readily be verified that none of them can be removed without enlarging the Study variety. The Hilbert polynomial of ${\mathcal {I}}$ is

$$\begin{aligned} H(x)= & {} \tfrac{1}{302400}x^{10} +\tfrac{1}{7560}x^9 +\tfrac{47}{20160}x^8 +\tfrac{1}{42}x^7 +\tfrac{2243}{14400}x^6\\&+\tfrac{49}{72}x^5 +\tfrac{121279}{60480}x^4 +\tfrac{2963}{756}x^3 +\tfrac{121883}{25200}x^2 +\tfrac{709}{210}x +1. \end{aligned}$$

We read off that the projective dimension of the Study variety equals $\dim \mathcal {S}= \deg H(x) = 10$. As expected, this equals $\dim {\text {SO}}(4,1) = 10$. Moreover, the degree of $\mathcal {S}$ as projective variety equals $\deg \mathcal {S}= \frac{1}{302400}\deg H(x)! = 12$.

The ideal (11) of $\mathcal {S}$ may seem unwieldy at first sight. Nonetheless, it has sufficient structure to allow for actual computations. We will encounter examples of this later in this text. We summarize our findings in

#### Theorem 1

The Study variety $\mathcal {S}\subset \mathbb {P}^{15}$ of conformal kinematics is given by the ideal (12) which is generated by ten bilinear polynomials. It is a projective variety of dimension ten and degree twelve.

### The Null Quadric

From (10) we see that the real parts of $q\widetilde{q}$ and $\widetilde{q}q$ are the same and vanish if and only if

13 $$\begin{aligned} q_0\widetilde{q_0} - S(q_1,q_2) - q_3\widetilde{q_3} = 0. \end{aligned}$$

This equation defines a regular quadric $\mathcal {N}\subset \mathbb {P}^{15} = \mathbb {P}({\text {CGA}} _+)$ which we call the *null quadric.* It extends the concept of the *null cone* $\mathcal {C}$ in the dual quaternion model of rigid body kinematics. Indeed, when plugging $q_2 = q_3 = 0$ into (13) we obtain $q_0\widetilde{q_0} = 0$, the equation of a singular quadric of rank four in $\mathbb {P}^7 = \mathbb {P}(\mathbb {D}\mathbb {H})$.

Denote the ideal of the “left” Study condition $q\widetilde{q} \in \mathbb {R}$ by ${\mathcal {L}}$ and the ideal of the “right” Study condition $\widetilde{q}q \in \mathbb {R}$ by ${\mathcal {R}}$, c.f. (10). Using computational algebraic geometry software, it can be shown that

$$\begin{aligned} {\mathcal {L}} = {\mathcal {I}} \cap {\mathcal {L}}',\quad {\mathcal {R}} = {\mathcal {I}} \cap {\mathcal {R}}' \end{aligned}$$

where ${\mathcal {L}}'$ and ${\mathcal {R}}'$ are irreducible ideals of dimension ten and degree 20 that both contain the polynomial defining the null quadric ${\mathcal {N}}$. This tells us that the “difference” between the “left” and the “right” Study variety is just in the null quadric $\mathcal {N}$. The non-null points of $\mathcal {S}$ are already determined by ${\mathcal {L}}$ or ${\mathcal {R}}$.

As already mentioned, points of $\mathcal {S}{\setminus } \mathcal {N}$ represent conformal displacements. The algebraic closure of this set is $\mathcal {S}$ while $\mathcal {S}\cap \mathcal {N}$ can be thought of as its “boundary”. The importance of boundaries in this sense for questions of kinematics, in general, has been demonstrated in several publications of the last decade, i.e. [2, 5, 7]. We will return to this in Sect. 4.

### Kinematic Groups and Subvarieties

Let us briefly explain how important groups of conformal and rigid body kinematics can be described in terms of the four quaternion representation $q = q_0 + q_1\varepsilon _1 + q_2\varepsilon _2 + q_3\varepsilon _3$ and the Study variety ideal ${\mathcal {I}}$ in the form (11).

#### Example 1

The *special orthogonal group* ${\text {SO}}(3)$ is encoded as ${\mathcal {I}} + \langle q_1, q_2, q_3 \rangle$. Because of bilinearity of the generators of ${\mathcal {I}}$ this simplifies to $\langle q_1, q_2, q_3 \rangle$. This just reconfirms the fact that ${\text {SO}}(3)$ is encoded by the quaternion group $\mathbb {H}$.

#### Example 2

The *group of rigid body displacements* ${\text {SE}}(3)$ is encoded as ${\mathcal {I}} + \langle q_2, q_3 \rangle$. This simplifies to the ideal $\langle S(q_0,q_1), q_2, q_3\rangle$ which is the well-known dual quaternion representation of ${\text {SE}}(3)$ [15, Chapter 11]. Composing ${\text {SE}}(3)$ with a fixed orientation reversing Euclidean displacement, for example the reflection in the origin, which is represented by $\varepsilon _3$, yields the *set* ${\text {E}}^{-}(3)$ *of all orientation reversing Euclidean displacements.* From

$$\begin{aligned} (q_0 + \varepsilon _1 q_1) \varepsilon _3 = q_1\varepsilon _1 + q_0\varepsilon _3 \end{aligned}$$

we see that ${\text {E}}^{-}(3)$ is represented by the ideal $\langle S(q_1,q_3), q_0, q_2 \rangle$.

#### Example 3

The *group* ${\text {Sim}}(3)$ *of direct similarities* is obtained by composing an element of ${\text {SE}}(3)$ with a uniform scaling. The scaling with factor $\sigma ^{-1/2}$ is given by the homogeneous spinor $s = 2 + (1-\sigma )e_{\infty o} = s_0 + \varepsilon _3 s_3$ where $s_0 = 1+\sigma$ and $s_3 = 1-\sigma$. Writing the rigid body displacement as $r = p + \varepsilon _1 d$ where $S(p,d) = 0$ we find

14 $$\begin{aligned} rs = (p + \varepsilon _1 d)(s_0 + \varepsilon _3 s_3) = ps_0 + d(s_0 + s_3)\varepsilon _1 + ps_3\varepsilon _3 = ps_0 + 2d\varepsilon _1 + ps_3\varepsilon _3.\nonumber \\ \end{aligned}$$

The ideal of ${\text {Sim}}(3)$ is generated as $\langle S(q_0,q_1), q_2, {{\,\mathrm{v}\,}}(q_0\widetilde{q_3}) \rangle$. The last condition encodes linear dependence of $q_0$ and $q_3$.

Note that neither ${\text {Sim}}(3)$ nor the group generated by ${\text {SO}}(3)$ and uniform scalings are represented by projective subspaces of the Study variety $\mathcal {S}$. This is different for the subgroup generated by scalings and translations:

#### Example 4

Denote by $v = v_1\mathbf {i}+ v_2\mathbf {j}+ v_3\mathbf {k}$ the translation vector. The composition of translation and scaling is obtained by plugging $p = 1$ and $d = -\frac{1}{2}v$ into (14), resulting in $s_0 - t\varepsilon _1 + s_3\varepsilon _3$. Thus, the ideal is

$$\begin{aligned} \langle {{\,\mathrm{v}\,}}(q_0), {{\,\mathrm{s}\,}}(q_1), q_2, {{\,\mathrm{v}\,}}(q_3) \rangle \end{aligned}$$

where ${{\,\mathrm{s}\,}}(\cdot )$ denotes the scalar part of a quaternion. This ideal is generated by eleven linear equations that indeed describe a projective subspace of dimension four.

#### Example 5

The composition of an inversion in the unit sphere, a translation, and another inversion in the unit sphere is called a *transversion* [4, Section 16.4]. More generally, we can replace the translation by an arbitrary element of ${\text {SE}}(3)$. This generates a subgroup isomorphic to ${\text {SE}}(3)$ whose elements have the four quaternion representation $q_0 + \varepsilon _2 q_2$. Transversions in the stricter sense of [4, Section 16.4] appear for $q_0 \in \mathbb {R}$.

## Straight Lines on the Study Variety

The kinematic interpretation of straight lines on the Study quadric $\mathcal {Q}$ is well-known [15, Section 11.2.1]. Straight lines through the identity displacement correspond, in general, to rotations around a fixed axis or, in exceptional cases, to translations in a fixed direction. General straight lines on $\mathcal {Q}$ correspond to rotations or translations, composed with a fixed rigid body displacement, either from the left or from the right. For a given straight line trough the identity displacement [1] we can chose an arbitrary point $[q_0+\varepsilon _1 q_1]\ne [1]$ with quaternions $q_0$, $q_1\in \mathbb {H}$ on the line to obtain a parametric equation $t+q_0+\varepsilon _1 q_1$. Here *t* is a real parameter, the point $[q_0+\varepsilon _1 q_1]$ is obtained for $t=0$ while [1] is obtained in the limit $t\rightarrow \infty$. The Study condition has to be fulfilled identically in *t* so that $q_1$ is necessarily vectorial. With $q = q_0 + \varepsilon _1 q_1$ this is equivalent to $q + \widetilde{q} \in \mathbb {R}$. It is no loss of generality to assume that $q_0$ is vectorial also, as otherwise we can re-parametrize via $t \mapsto t - {{\,\mathrm{s}\,}}(q_0)$. This can be encoded as $q + \widetilde{q} = 0$. In this section we generalize these relations to ${\text {CGA}} _+$ and its Study variety $\mathcal {S}$.

The cases of rotations and translations can be distinguished by the position of the straight line with respect to the null cone $\mathcal {C}$. Straight lines corresponding to rotations intersect $\mathcal {C}$ in a pair of conjugate complex points while straight lines corresponding to translations intersect in the point $[\varepsilon _1q_1]$ with multiplicity two. As we shall see, the classification of straight lines in the Study variety $\mathcal {S}$ via the number of their real intersection points with the null quadric $\mathcal {N}$ is natural in ${\text {CGA}} _+$ as well.

We proceed with investigating straight lines through the identity displacement and contained in $\mathcal {S}$. In the light of above discussion, obvious examples come from rotations around a fixed axis and translations in a fixed direction. They are special cases of simple conformal motions described by Dorst in [3]. Slightly adapting Dorst’s Equation (2), we can define:

### Definition 2

A *simple motion* is given by the exponential $\mathrm {e}^{uq}$, where *u* is a real parameter and *q* is a 2-blade, that is $q = a \wedge b$, where *a* and *b* are vectors. This exponential is given by

15 $$\begin{aligned} \mathrm {e}^{uq} = {\left\{ \begin{array}{ll} \cos u + q \sin u &{}\quad \text {if } q\widetilde{q} > 0, \\ 1 + q u \quad &{}\quad \text {if } q\widetilde{q} = 0, \\ \cosh u + q \sinh u \quad &{}\quad \text {if } q\widetilde{q} < 0. \end{array}\right. } \end{aligned}$$

We claim that simple motions give examples of straight lines in $\mathcal {S}$:

Writing $a = a_o e_o + a_\infty e_\infty + a_1e_1 + a_2e_2 + a_3e_3$, $b = b_o e_o + b_\infty e_\infty + b_1e_1 + b_2e_2 + b_3e_3$ and define $q_a \,{:}{=}\,a_1\mathbf {i}+ a_2\mathbf {j}+ a_3\mathbf {k}$, $q_b \,{:}{=}\,b_1\mathbf {i}+ b_2\mathbf {j}+ b_3\mathbf {k}$. Replacing *a* or *b* by a linear combination of *a* and *b* changes *q* only up to an irrelevant real factor. Thus, it is no loss of generality to assume $b_o = 0$. We then have

16 $$\begin{aligned} q = a \wedge b = -q_a \times q_b + (b_\infty q_a-a_\infty q_b) \varepsilon _1 - a_o q_b \varepsilon _2 - a_o b_\infty \varepsilon _3. \end{aligned}$$

We see that *q* has the four quaternion representation $q = q_0 + q_1\varepsilon _1 + q_2\varepsilon _2 + q_3\varepsilon _3$ with ${{\,\mathrm{s}\,}}(q_0) = {{\,\mathrm{s}\,}}(q_1) = {{\,\mathrm{s}\,}}(q_2) = {{\,\mathrm{v}\,}}(q_3) = 0$. It is easy to verify that the point [*q*] satisfies the Study conditions (12). Any of the parametric equations (15) is equivalent to a parametric equation of type $t + q$ via a simple parameter transformation (either $t = \cot u$, $t = u^{-1}$, or $t = \pm \coth u$). This is, indeed, the parametric equation for a straight line through the identity displacement. It is contained in $\mathcal {S}$ by Dorst’s construction (or by Theorem 2 below). Note that the identity displacement [1] is only obtained in the limit for $\vert t \vert \rightarrow \infty$.

The main insight of this section is that all straight lines $t + q$ through the identity and contained in $\mathcal {S}$ are simple motions. We proceed by deriving the conditions on *q* that ensure that $[t+q] \in \mathcal {S}$ for any $t \in \mathbb {R}$ and then demonstrate that these conditions allow the decomposition $q = a \wedge b$ with vectors *a* and *b*.

### Theorem 2

The straight line $t + q$ with $q = q_0 + q_1\varepsilon _1 + q_2\varepsilon _2 + q_3\varepsilon _3$ is contained in the Study variety $\mathcal {S}$ if and only if $[q] \in \mathcal {S}$ and $q + \widetilde{q} \in \mathbb {R}$. If ${{\,\mathrm{s}\,}}(q_0) = 0$, then this is equivalent to $q + \widetilde{q} = 0$.

### Proof

Recall that $[q] \in \mathcal {S}$ is equivalent to $q\widetilde{q} = \widetilde{q}q \in \mathbb {R}$. Now, $(t+q)(t+\widetilde{q})$ and $(t+\widetilde{q})(t+q)$ need to be real for any $t \in \mathbb {R}$. From

$$\begin{aligned} (t+q)(t+\widetilde{q}) = (t+\widetilde{q})(t+q) = t^2 + (q + \widetilde{q})t + \widetilde{q}q \end{aligned}$$

we infer that this is the case if and only if $q + \widetilde{q}$ and $\widetilde{q}q$ is real. The latter condition implies $[q] \in \mathcal {S}$ and the theorem’s first claim follows. The second can be read off from the explicit representation

17 $$\begin{aligned} \frac{1}{2}(q + \widetilde{q}) = {{\,\mathrm {s}\,}}(q_0) + {{\,\mathrm {s}\,}}(q_1)\varepsilon _1 + {{\,\mathrm {s}\,}}(q_2)\varepsilon _2 + {{\,\mathrm {v}\,}}(q_3)\varepsilon _3. \end{aligned}$$

$$\square $$

### Lemma 1

If $q + \widetilde{q} = 0$ there exist vectors *a*, *b* such that $q = a \wedge b$.

### Proof

The case $q = 0$ is trivial and not of interest to us. We will exclude it for the remainder of this proof.

With $q \ne 0$ given as $q = q_0 + q_1\varepsilon _1 + q_2\varepsilon _2 + q_3\varepsilon _3$, (16) implies that we have to solve

18 $$\begin{aligned} q_0 = -q_a \times q_b,\quad q_1 = b_\infty q_a-a_\infty q_b,\quad q_2 = - a_o q_b,\quad q_3 = - a_o b_\infty \end{aligned}$$

for the scalars $a_0$, $a_\infty$, $b_\infty$ and the vectors $q_a$ and $q_b$. From (17) we see that $q_0$, $q_1$, and $q_2$ are vectorial and $q_3$ is scalar. This is an obvious necessary condition for (18) to have a solution. We distinguish the two cases $q_3 = 0$ and $q_3 \ne 0$.

Case 1: $q_3 = 0$: If $q_2 = 0$ (Case 1.1; rigid body displacements), then either $a_o = 0$ or $b_\infty = q_b = 0$. The latter implies $b = 0$ and is not possible because we assumed $q \ne 0$. Thus, $a_o = 0$. Now, we are left with the two equations

$$\begin{aligned} q_0 = -q_a \times q_b,\quad q_1 = b_\infty q_a-a_\infty q_b. \end{aligned}$$

We view them as vector equations in $\mathbb {R}^3$. If $q_0 = 0$, we can pick $q_a$ and $q_b$ as scalar multiples of $q_1$ to satisfy the first equation and then solve the second equation for $a_\infty$ and $b_\infty$. If $q_0 \ne 0$, the first equation admits infinitely many solutions for $q_a$ and $q_b$, all of them in the orthogonal complement $q_0^\perp$ of $q_0$ and linearly independent. By the Study conditions on *q* we have $S(q_0,q_1) = 0$ so that $q_1 \in q_0^\perp$ as well. Therefore, given solutions $q_a$ and $q_b$ of the first equation, the second equation can be solved for $a_\infty$ and $b_\infty$.

If $q_2 \ne 0$ (Case 1.2), then $a_o \ne 0$ so that $b_\infty = 0$. We set $a_o = -1$ and $q_b = q_2$ to satisfy the third equation of (18). The remaining equations are

$$\begin{aligned} q_0 = -q_a \times q_2,\quad q_1 = -a_\infty q_2. \end{aligned}$$

By (11), points of the Study variety satisfy ${{\,\mathrm{v}\,}}(q_1\widetilde{q_2})-{{\,\mathrm{v}\,}}(q_0\widetilde{q_3}) = 0$ which simplifies to $q_1q_2 \in \mathbb {R}$, or equivalently $q_1 \times q_2 = 0$, in our case. Thus, $q_1$ and $q_2$ are linearly dependent as vectors in $\mathbb {R}^3$. Since $q_2 \ne 0$, the second equation can be solved uniquely for $a_\infty$ while the first equation admits infinitely many solutions for $q_a$.

Case 2: $q_3 \ne 0$. We set $a_o = -1$, $b_\infty = q_3$, and $q_b = q_2$ to satisfy the third and fourth equation of (18). The two remaining equations read

$$\begin{aligned} q_0 = -q_a \times q_2, \quad q_1 = q_3q_a - a_\infty q_2. \end{aligned}$$

Again, we view them as vector equations in $\mathbb {R}^3$. Solving the second equation for $q_a$ yields $q_a = (q_1 + a_\infty q_2)/q_3$. Plugging this into the first equation leads to $q_0q_3 = -q_1 \times q_2$. This holds true as it is only an alternative way of writing the condition ${{\,\mathrm{v}\,}}(q_0\widetilde{q_3}) - {{\,\mathrm{v}\,}}(q_1,\widetilde{q_2}) = 0$ from (11).

After having discussed all possible cases and sub-cases, the proof is complete. $\square$

### Theorem 3

Simple motions correspond to straight lines on $\mathcal {S}$ through the identity displacement [1] and vice versa.

### Proof

We have already argued that a simple motion can be parameterized as $t + q$ where $[q] \in \mathcal {S}$ and $q + \widetilde{q} = 0$. This is, indeed, a straight line through [1]. Conversely, given a straight line on $\mathcal {S}$ and through [1] it can always be parametrized as $t + q$ with $q + \widetilde{q} \in \mathbb {R}$ by Theorem 2. The admissible re-parametrization $t \mapsto t - {{\,\mathrm{s}\,}}(q_0)$ then implies $q + \widetilde{q} = 0$ whence $q = a \wedge b$ with vectors *a*, *b* by Lemma 1. But then $t + q$ is a simple motion by Definition 2. $\square$

The decomposition $q = a \wedge b$ of Lemma 1 is not unique. Replacing vectors *a* with the linear combination $\alpha _1 a+\beta _1 b$ and *b* by $\alpha _2 a+\beta 2 b$, for $\alpha _1$, $\alpha _2$, $\beta _1$, $\beta 2\in \mathbb {R}$ with $\alpha _1\beta _2-\alpha _2\beta _1=1$ will result in the same *q*. By taking independent linear combinations of *a* and *b*, we will obtain a scalar multiple of *q*, which represents the same point on the Study variety. General vectors *a* and *b* represent spheres in ${\text {CGA}}$. The possibility to pick special spheres, i.e., planes or points, in the decomposition $q = a \wedge b$ of *q* gives rise to six types of simple motions [3]: conformal rotations, conformal scalings, transversions, rotations, translations and uniform scalings.

In our context, it is maybe more natural to primarily distinguish three types of straight lines $t + q$ through the identity displacement and on $\mathcal {S}$ according to the number *n* of real intersection points with the null quadric $\mathcal {N}$. They correspond to the Dorst cases conformal rotation ($n = 0$), transversion ($n = 1$) in a more general sense than in Example 5, and conformal scaling ($n = 2$). Sub-cases with special Euclidean relevance are Euclidean rotation ($n = 0$), translation ($n = 1$), and uniform scaling ($n = 2$). They are easy to distinguish in the four quaternion representation of $q = q_0 + q_1\varepsilon _1 + q_2\varepsilon _2 + q_3\varepsilon _3$, c.f. Examples 2 and 4:

- Euclidean rotations among conformal rotations and translations among transversions are characterized by $q_2 = q_3 = 0$, that is, they are rigid body displacements.
- Uniform scalings are characterized among conformal scalings by ${{\,\mathrm{v}\,}}(q_0) = {{\,\mathrm{s}\,}}(q_1) = q_2 = {{\,\mathrm{v}\,}}(q_3) = 0$. The condition ${{\,\mathrm{s}\,}}(q_1) = 0$ is already implied by the fact that $t + q$ is contained in the Study variety $\mathcal {S}$.

The six simple motions can also be distinguished by the number $f \in \{0,1,2\}$ of their real fixpoints and, if $f \ge 1$, whether $e_\infty$ is among them. The relation of fixpoints to the intersection points of $t + q$ with $\mathcal {N}$ is investigated next.

### Conformal Rotations and Conformal Scalings

Given a straight line $t + q$ on $\mathcal {S}$ and through the point [1] we already argued (Lemma 1) that *q* can be chosen as $q = a \wedge b$ with vectors *a*, *b*. In the two generic cases of Dorst’s classification (conformal rotation and conformal scaling), *a* and *b* are two points — conjugate complex in case of conformal rotations and real in case of conformal scalings. We compute the parameter values $t_1$, $t_2$ of the intersection points $[n_1]$, $[n_2]$ with the null quadric $\mathcal {N}$. From

$$\begin{aligned} (t + q)(t + \widetilde{q}) = (t + q)(t - q) = t^2 - q^2 = t^2 - (a \cdot b)^2 \end{aligned}$$

we infer $t_1 = a \cdot b$ and $t_2 = -a \cdot b$ and hence $[n_1] = [ab]$, $[n_2]=[-ba]=[ba]$.

Consider now, at least formally, the $n_1$-image of a point *x*. We compute

$$\begin{aligned} y = n_1x\widetilde{n_1} = 4 (a \cdot b)(b \cdot x) a \end{aligned}$$

so that the $n_1$-image of a point *x* equals *a* unless $b \cdot x = 0$, i.e., *x* is “perpendicular” to *b*. In this sense, the “null displacement” $n_1$ has the generic image point *a*. The generic image point for $n_2$ is *b*.

### Uniform Scalings

A similar computation is possible for $q = a \wedge b$ where *a* represents the point with coordinates $(a_1,a_2,a_3)$ and $b = e_\infty$. According to [3], this is a uniform scaling with center *a* (an “isotropic” scaling, as it is called there). We have

$$\begin{aligned} q = a_1e_{1\infty } + a_2e_{2\infty } + a_3e_{3\infty } - e_{\infty o} - 1 \quad \text {and}\quad (t + q)(t + \widetilde{q}) = t^2 - 1 \end{aligned}$$

so that the intersection points with $\mathcal {N}$ are

$$\begin{aligned} \left[ n_1\right]= & {} \left[ -1+q\right] = \left[ a_1e_{1\infty } + a_2e_{2\infty } + a_3e_{3\infty } - e_{\infty o} - 2\right] = \left[ ae_\infty \right] ,\\ \left[ n_2\right]= & {} \left[ 1+q\right] = \left[ a_1e_{1\infty } + a_2e_{2\infty } + a_3e_{3\infty } - e_{\infty o}\right] = \left[ -\widetilde{n_1}\right] = \left[ \widetilde{n_1}\right] . \end{aligned}$$

Acting with $n_1$ and $n_2$ on a generic point *x* gives

$$\begin{aligned} n_1x\widetilde{n_1} = 4a, \quad n_2x\widetilde{n_2} = -4(a \cdot x) e_\infty , \end{aligned}$$

respectively. We see that the action of $n_1$ on *x* always gives point *a*. The only exception is $x = e_\infty$ where the action is undefined due to $n_1e_\infty \widetilde{n_1} = 0$. The action of $n_2$ on *x* gives $e_\infty$ unless $a \cdot x = 0$.

A more elementary interpretation for $n_1$ and $n_2$ is scalings with center *a* and respective scaling factors 0 and $\infty$. It is very intuitive to say that the zero-scaling $n_1$ fixes $e_\infty$ and maps everything else to *a*. Likewise, the $\infty$-scaling $n_2$ fixes *a* and maps everything else to $e_\infty$.

### Transversions and Translations

A transversion is given by $q = a \wedge b$ where *a* represents a point and *b* is a plane perpendicular to *a*. If $a \ne e_\infty$ we may write $a = (a_1,a_2,a_3)$ whence

$$\begin{aligned} b = b_1 e_1 + b_2 e_2 + b_3 e_3 + (a_1 b_1 + a_2 b_2 + a_3 b_3) e_\infty . \end{aligned}$$

Because of $a \cdot b = 0$ we have $q = a \wedge b = ab$. Because *a* and *b* are both vectors, we further have $\widetilde{q}= ba$ so that $q\widetilde{q} = \widetilde{q}q = abba = a^2b^2 = 0$, because $a^2 = 0$. By (16) $q + \widetilde{q} = 0$ so that $(t+q)(t+\widetilde{q}) = t^2$ whence *q* is, indeed, the only intersection point of the straight line $t+q$ with $\mathcal {N}$. Acting on a generic point *x* yields

$$\begin{aligned} qx\widetilde{q} = -2 b^2 (a \cdot x) a. \end{aligned}$$

We infer that *a* is the image of point *x* unless *x* is perpendicular to *a*. A similar computation is also possible for $a = e_\infty$. It gives a translation.

Summarizing our findings in Sects. 4.1–4.3, we can say that a simple motion, represented by a straight line $t + q$ on $\mathcal {S}$ and through the point [1] has up to two exceptional real points that are obtained as images of a generic point under the displacements obtained as real intersection points of $t + q$ with the null quadric $\mathcal {N}$. They are fixed by the motion but the set of fixed points is generically larger.

#### Remark 1

Euclidean rotations are somewhat exceptional in this context. Here $q = a \wedge b$ with *planes* *a* and *b*. In the projective closure of Euclidean three-space, one might be tempted to consider planes with $a^2 = b^2 = 0$ as complex points at infinity. This viewpoint is uncommon in the conformal closure of Euclidean three-space.

## Outlook

An important motivation for our study is extension of the factorization theory of motion polynomials [6, 9, 10] to “spinor polynomials” of conformal geometric algebra. These are defined as univariate polynomials *C* with coefficients in ${\text {CGA}} _+$ such that $C\widetilde{C} = \widetilde{C}C$ is a real polynomial different from 0. They describe conformal motions where all point trajectories are rational curves. Factorization of *C* into linear factors corresponds to the decomposition of the conformal motion into concatenated simple motions. Results of [9] essentially imply that such factorizations do exist for “generic” spinor polynomials. Typically, they are not unique. We present one example:

### Example 6

The polynomial $C = (t + h_1)(t + h_2)(t + h_3)$ with

$$\begin{aligned} h_1 = - e_{1\infty } + 2e_{1o},\quad h_2 = - e_{2\infty } - 2e_{2o},\quad h_3 = 1 - \tfrac{1}{2} e_{3\infty } + e_{3o} + e_{\infty o} \end{aligned}$$

satisfies $$C\widetilde{C} = M_1 M_2 M_3$$ where

$$\begin{aligned} \begin{aligned} M_1&\,{:}{=}\,(t + h_1)(t + \widetilde{h}_1) = t^2 + 4,\\ M_2&\,{:}{=}\,(t + h_2)(t + \widetilde{h}_2) = t^2 - 4,\\ M_3&\,{:}{=}\,(t + h_3)(t + \widetilde{h}_3) = t^2. \end{aligned} \end{aligned}$$

It is thus a spinor polynomial. We see that it is the composition of a transversion, a conformal scaling, and a conformal rotation. They correspond, in that order, to $h_3$, $h_2$, and $h_1$. Using Algorithm 2 of [9] we can compute further factorizations $C = (t+k_1)(t+k_2)(t+k_3) = (t+\ell _1)(t+\ell _2)(t+\ell _3)$ where

$$\begin{aligned} \begin{aligned} k_1&= -e_{2\infty } - 2 e_{2 o},\\ k_2&= -1 + \tfrac{1}{2} e_{3\infty } - e_{3 o} - e_{\infty o},\\ k_3&= \quad 2 - e_{1\infty } + 2 e_{1 o} - e_{3\infty } + 2 e_{3 o} + 2 e_{\infty o}, \end{aligned} \end{aligned}$$

and

$$\begin{aligned} \begin{aligned} \ell _1&= \quad 1 + e_{2 3} - \tfrac{1}{2} e_{2 \infty } - e_{2 o} + \tfrac{1}{2} e_{3 \infty } + e_{3 o},\\ \ell _2&= -2 - e_{2 3} - \tfrac{1}{2} e_{2 \infty } - e_{2 o} - 2 e_{3 o} - e_{\infty o},\\ \ell _3&= \quad 2 - e_{1 \infty } + 2 e_{1 o} - e_{3 \infty } + 2 e_{3 o} + 2 e_{\infty o}. \end{aligned} \end{aligned}$$

Because of $(t+k_1)(t+\widetilde{k}_1) = M_2$, $(t+k_2)(t+\widetilde{k}_2) = M_3$, and $(t+k_3)(t+\widetilde{k}_3) = M_1$ the factors $k_3$, $k_2$, and $k_1$ correspond, in that order, to a conformal rotation, a transversion, and a conformal scaling. Finally, we have $(t+\ell _1)(t+\widetilde{\ell }_1) = t(t+2)$, $(t+\ell _2)(t+\widetilde{\ell }_2) = t(t-2)$, $(t+\ell _3)(t+\widetilde{\ell }_3) = M_1$, the third factorization correspond to the decomposition into a conformal rotation and two conformal scalings.$\diamond$

Algorithm 2 of [9] produces indeed, the maximal number of twelve factorizations of the polynomial *C* of Example 6. Each of these factorizations corresponds to one of the twelve ways of writing $C\widetilde{C}$ as product of real quadratic polynomials. The algorithm relies on the fact that certain algebra elements, obtained as leading coefficients of linear remainders of polynomial division, are invertible. This is generic behavior but non-generic examples are assumed to exist. Factorizability results in this sense will be the topic of a future publication.

## Conclusion

We have introduced Study variety $\mathcal {S}$ and null quadric $\mathcal {N}$ of conformal kinematics and investigated some of their properties. The structure of their ideals becomes clearer in our four quaternion representation of ${\text {CGA}} _+$. We also use the four quaternion representation for investigating straight lines on $\mathcal {S}$. They turn out to be related to elementary motions suggested by Dorst in [3].

We view our results as generalizations of the representation of ${\text {SE}}(3)$, the groups of rigid body displacements, where dual quaternions give rise to a kinematic from ${\text {SE}}(3)$ to the points of the Study quadric $\mathcal {Q}$ minus the null cone $\mathcal {C}$. Straight lines on $\mathcal {Q}$ are known to correspond to rotations around a fixed axis or translations in a fixed direction. They appear naturally in the factorization theory of motion polynomials [6] and we have pointed at similar relations between “spinor polynomials” and straight lines on $\mathcal {S}$, that is, simple motions.
